# Establishment of CDC Global Rapid Response Team to Ensure Global Health Security

**DOI:** 10.3201/eid2313.170711

**Published:** 2017-12

**Authors:** Tasha Stehling-Ariza, Adrienne Lefevre, Dinorah Calles, Kpandja Djawe, Richard Garfield, Michael Gerber, Margherita Ghiselli, Coralie Giese, Ashley L. Greiner, Adela Hoffman, Leigh Ann Miller, Lisa Moorhouse, Carlos Navarro-Colorado, James Walsh, Dante Bugli, Cyrus Shahpar

**Affiliations:** Centers for Disease Control and Prevention, Atlanta, Georgia, USA

**Keywords:** Centers for Disease Control and Prevention, CDC, Global Rapid Response Team, public health, epidemiology, emergency response, global health security, outbreak, disaster, rapid response, Ebola, viruses

## Abstract

The 2014–2016 Ebola virus disease epidemic in West Africa highlighted challenges faced by the global response to a large public health emergency. Consequently, the US Centers for Disease Control and Prevention established the Global Rapid Response Team (GRRT) to strengthen emergency response capacity to global health threats, thereby ensuring global health security. Dedicated GRRT staff can be rapidly mobilized for extended missions, improving partner coordination and the continuity of response operations. A large, agencywide roster of surge staff enables rapid mobilization of qualified responders with wide-ranging experience and expertise. Team members are offered emergency response training, technical training, foreign language training, and responder readiness support. Recent response missions illustrate the breadth of support the team provides. GRRT serves as a model for other countries and is committed to strengthening emergency response capacity to respond to outbreaks and emergencies worldwide, thereby enhancing global health security.

The need to detect and respond to disease outbreaks before they spread has long been recognized as a priority because uncontained outbreaks can rapidly proliferate into international emergencies (*1*–*3*). A jarring example was provided by the 2014–2016 Ebola virus disease (Ebola) epidemic in West Africa, in which ≈29,000 cases were identified and ≈11,000 patients died (*1*,*4*). Although most cases occurred in 3 countries, imported and locally transmitted cases were confirmed in 7 others, including the United States (*5*). This experience highlighted needs for improved international collaboration and coordination and stronger national response capacity to rapidly detect and control major health threats at their source to ensure global health security (*3*,*6*–*10*).

The 2005 International Health Regulations (IHR 2005), adopted by the World Health Organization, dictate that all member states should be prepared to detect and respond to public health threats and emergencies (*11*). However, by 2012, <20% of countries reported full compliance with IHR 2005 (*12*). To accelerate progress, several member states and international partners launched the Global Health Security Agenda, which outlines specific actions that countries can take to meet IHR 2005 requirements (*6*,*7*,*13*–*15*). The US Centers for Disease Control and Prevention (CDC), in coordination with other US government agencies and global partners, is using its expertise and the Global Health Security Agenda framework to assist partner countries and strengthen global health security (*16*).

CDC has a long history of responding to global public health emergencies, including polio and severe acute respiratory syndrome. It is internationally recognized for its expertise in disease detection, investigation, diagnosis, monitoring, and control, as well as management of public health emergencies (*16*). Several groups within CDC work closely to identify and respond to public health threats. The Global Disease Detection Operations Center (GDDOC) is dedicated to the detection and monitoring of global public health events of international importance (*17*). GDDOC links external requests for assistance with the appropriate disease-specific CDC subject matter experts, who respond frequently to domestic and international outbreaks of diseases in their program domains. GDDOC also serves as an agency liaison to the Global Outbreak Alert and Response Network (GOARN) and supports the mobilization of subject matter experts through GOARN. In the field, responders work closely with governments and partners, including within Incident Management System structures or health clusters when established. Although mobilized CDC responders do not provide medical care, such activities are coordinated with organizations providing patient care.

Before the Ebola epidemic, when response operations exceeded subject matter expert program capacity, surge staff from the Epidemic Intelligence Service and other CDC programs were engaged and coordinated by the CDC Division of Emergency Operations (DEO). For larger, complex public health responses, the CDC director can authorize the activation of an agency-level Incident Management System, supported by the CDC emergency management subject matter experts in DEO and ordinarily based in the CDC Emergency Operations Center (EOC) (*18*). DEO also provides logistical and other support to response operations funded by GDDOC without activating the Incident Management System. At the time of the Ebola epidemic, CDC lacked a formal pool of on-call, trained responders who could rapidly mobilize for extended periods and in large numbers.

In July 2014, CDC activated its Incident Management System in response to the Ebola epidemic; as the largest agencywide response ever, it tested the limits of the agency response capacity (*19*). During July 9, 2014–March 31, 2016, ≈4,000 CDC staff participated in the response in Ebola-affected countries; in countries at high risk for Ebola introduction; from CDC headquarters in Atlanta, Georgia, USA; or through other partner organizations (*1*). By March 31, 2016, CDC had supported ≈2,000 mobilizations of 1,400 personnel providing wide-ranging technical support, for ≈80,000 person-days of mobilization time (*19*–*23*).

The size, scale, severity, and duration of the Ebola response highlighted key challenges to the efficiency and effectiveness of international emergency response efforts (Table). Specifically, greater support from the international community was needed because of limited national capacity of affected countries to detect and respond to the outbreak, fundamental aspects of IHR 2005, and the diminishing healthcare capacity over the course of the epidemic (*1*). Despite CDC experience regularly providing assistance for smaller, shorter outbreaks, sustaining support over 21 months proved difficult. Because of limited CDC presence before the epidemic, weak or underdeveloped relationships with governments and partner organizations in affected countries hindered response coordination. Short mobilizations (typically 30 days) and frequent staff rotation in the field also disrupted development of long-standing relationships and continuity of response. However, longer mobilizations of such a large workforce could hamper staff members’ regular duties, potentially affecting other CDC programs (*1*,*19*,*20*). Additional challenges included identifying staff with the appropriate technical skills and foreign language abilities who were mentally and emotionally prepared for the austere conditions and ready and available to mobilize (*19*,*20*).

**Table:** Challenges encountered during response to the 2014–2016 Ebola epidemic in West Africa and GRRT mitigation strategies

Challenge	GRRT strategy
Limited in-country capacity to detect and respond to disease outbreaks (*1*)	Support the development of national outbreak detection and response systems
Wide range of technical expertise required to address needs of a large outbreak response (*1*)	Recruit team members with a wide range of technical expertise and experience
	Train responders in multiple technical areas for high-risk diseases
Establishing working partnerships with governments and partner organizations for more efficient coordination (*1**,**19**,**20*)	Train responders on working with partner organizations, incident management systems, cultural sensitivity, and foreign languages
	Recruit dedicated, ready responders who can mobilize for up to 6 mo for stronger partner relationships and improved coordination
Short mobilizations (traditionally 30 d) and frequent rotation of staff disrupted continuity of response activities (*19**,**20*)	Recruit dedicated responders who are available and ready to mobilize for up to 6 mo if needed
	Expand the typical mobilization length of those in leadership roles
	Develop best practices and systems for information management in field response
Responder preparation and readiness (*19*)	Strengthen safety, security, and responder wellness training through a GRRT orientation
	Support continuous learning by offering frequent technical trainings on priority topics
	Track responder international travel–related mobilization requirements, training, and clearance compliance
	Obtain supervisor preapproval for mobilizations during on-call months
Identifying appropriate responders (*19*)	Roster GRRT responders and tracking skills and experience to match staffing needs
Limited foreign language capacity (*20*)	Develop a program to develop and validate foreign language capacity
Logistical support for field efforts (*19*)	Roster a group of dedicated and surge logisticians who can mobilize to provide support directly to responders in the field or coordinate with Atlanta-based logistics personnel to provide support

*GRRT, Global Rapid Response Team.

The challenges observed during the Ebola response underscored the need for a cadre of highly trained and experienced personnel who can rapidly mobilize to respond for extended periods (*20*). To address these challenges, CDC established the Global Rapid Response Team (GRRT). We describe the establishment of GRRT, team structure, main activities, case studies, and lessons learned.

## Establishment of GRRT

Before the Ebola epidemic ended, CDC began investing in its capacity to rapidly respond to public health emergencies. In June 2015, CDC launched GRRT to address many of the challenges recognized during the Ebola response and to support other countries when their national response capacity is overwhelmed. Housed within the Emergency Response and Recovery Branch (ERRB), Division of Global Health Protection, at the CDC Center for Global Health, GRRT is an agencywide asset mandated to strengthen emergency response capacity. GRRT stands ready to provide technical and nontechnical support for public health responses worldwide; it is the result of collaboration across CDC.

## GRRT Team Structure

GRRT comprises a small group of dedicated responders and a large group of agencywide surge staff. This model enables effective response to common events with a small number of experts while the team prepares for larger, rare events that necessitate substantial response. A total of 18 dedicated responders with public health emergency response expertise can immediately mobilize and remain in the field for extended periods. Included on this Atlanta-based team are multilingual epidemiologists with expertise in public health and humanitarian emergencies, logisticians who support GRRT activities and coordinate with DEO during a response, highly experienced team leaders, and support staff. Outside Atlanta, 1 regional emergency advisor in West Africa is tasked with engaging national, regional, and global partners to build capacity to detect and respond to health threats in the region. This group of dedicated responders answers the need to improve response time for emergencies, establish stronger long-standing relationships with governments and key partners, and reduce disruption to the continuity of response activities from staff turnover in the field.

GRRT surge capacity comprises >400 CDC staff members from around the agency; the goal is to support an emergency response with up to 50 staff members on short notice. Nearly 40 of the surge staff members routinely respond to humanitarian emergencies and build public health capacity as part of their regular duties in ERRB. They provide expertise in nutrition, emergency preparedness, surveillance, mental health, reproductive health, water, sanitation, and hygiene. The remaining surge staff vary widely in technical, language, and leadership skills and experience levels. They were recruited from 15 CDC centers, field personnel staff with state and local health departments, and overseas offices. International experience of the surge staff is a median of 2 years (mean 5 years), totaling 1,577 years combined. More than half have emergency response experience and ≈13% report having expertise in >1 foreign language. The most common occupations are epidemiologist, health scientist, public health advisor, and health communicator; surge staff have experience in nearly 30 different occupational areas.

Balancing the need to mobilize large numbers of agency staff, thereby possibly hindering their regular duties, with the need to ensure that existing programs maintain their operations is challenging (*19*). To address both needs, surge staff are on call 2 months each year for emergency mobilizations. The assignment of these on-call months is determined by staff availability (avoiding months in which regular duties or personal needs require the staff to be in the home office) while evenly distributing the technical skills, foreign language, and experience levels across months. The resulting roster lists at least 50 surge staff with a similar distribution of skills and experience who are on call for mobilization each month.

## GRRT Activities

Requests for assistance come from within CDC and from external partners. After receipt, requests are evaluated to determine the appropriate response mechanism. Requests meeting specific criteria are addressed through standard response mechanisms (e.g., GDDOC or subject matter expert mobilizations). GRRT reviews requests that do not meet the criteria or exceed capacity of other CDC groups. Decisions to respond are based on, among other considerations, the urgency, public health impact, and availability of appropriate staff to fill the request. After the decision to respond is made, responders are selected according to their skills, experience, and availability.

From September 1, 2015, through December 31, 2016, GRRT responders were mobilized 291 times for 10,148 person-days to work in 35 countries, territories, and the CDC EOC (Figure). Most of the mobilization time was spent responding to outbreaks of Zika virus infection (65.0%), yellow fever (9.4%), Ebola (4.3%), cholera (3.9%), polio (0.5%), and measles (0.5%). The remaining time went to natural disasters (Hurricane Matthew [12.8%] and wildfires in Indonesia [3.2%]). Responders aligned themselves with existing response activities, working directly with ministries of health, the World Health Organization (WHO), CDC country offices, and other partners.

**Figure:**
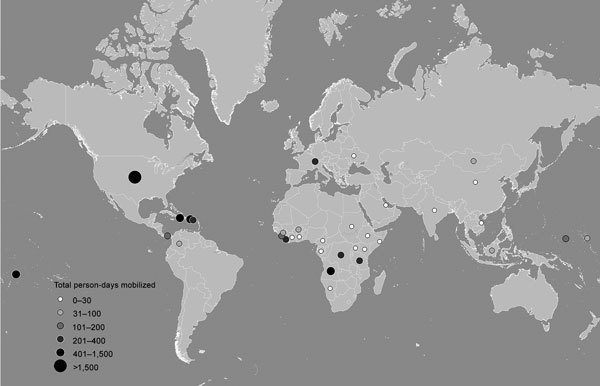
Global Rapid Response Team personnel mobilizations, September 2015–December 2016.

In addition to response activities, GRRT collaborated with ministries of health and external partners, such as the Africa CDC and the West African Health Organization, to assess and build national and international capacity to detect and respond to health threats, improving IHR compliance (320 person-days mobilized; median mobilization length 9 days). Activities included supporting the WHO Joint External Evaluations (*24*), developing rapid response team guidance, and facilitating response-related trainings.

Within CDC, GRRT works to build a sustainable, trained workforce. GRRT has designed a comprehensive training curriculum for surge staff that includes safety, security, soft skills, and technical training. GRRT increases responder readiness for rapid mobilization by defining and tracking training and logistical criteria. Continuing education is provided monthly for additional training opportunities beyond the baseline training received during a 1-day orientation. These trainings are hosted by subject matter expert groups throughout the agency and feature a combination of scientific topics, role-specific technical content for the field, interpersonal skills, and situational awareness updates depending on current emergency context. GRRT is also developing training focused on the principles of field team leadership in international response; the aim is preparing leaders to apply Incident Management System principles during mobilization while navigating the nuances of international field response. To enhance the agency’s foreign language capacity, GRRT provides foreign language training opportunities online and in classrooms. Efforts to standardize foreign language testing are under way.

## Case Studies

To illustrate the breadth of GRRT’s response work and its influence on agency response capacity, we describe selected responses to the Zika virus epidemic, urban outbreaks of yellow fever, and Hurricane Matthew in Haiti. Case studies demonstrate GRRT ability to support large complex outbreak responses, fill response needs when CDC expert capacity is strained, and manage smaller responses without EOC activation.

### 2015–2016 Zika Virus Response

In May 2015, an outbreak of Zika virus disease was reported in Brazil. In October, unusually high rates of birth defects, particularly microcephaly, were reported in areas with Zika virus transmission (*25*). By January 2016, Zika virus had spread to 14 countries and territories in Latin America and the Caribbean, and CDC activated an Incident Management System to respond to the outbreak (*26*). To support the response and address external requests for assistance, GRRT coordinated closely with subject matter experts, GDDOC, and DEO. 

The complex Zika virus response, with its expansive affected geographic area and multidisciplinary technical needs, tested the CDC emergency response capacity soon after the Ebola experience. Investigations into the modes of transmission, birth defects associated with infection, and effective interventions required subject matter experts in vectorborne diseases, maternal and child health, reproductive health, and birth defects. Laboratorians strengthened Zika virus testing capacity and improved existing diagnostic tools. Health communication specialists developed messages in multiple languages for varied audiences, balancing the relatively mild symptoms of infection experienced by most persons with the devastating consequences of infection during pregnancy (*26*).

GRRT supported the agency response by mobilizing 117 responders to 9 countries and territories for 151 mobilizations and 6,597 person-days. A total of 69 mobilizations and more than half of the response time (3,556 person-days) were in the CDC EOC, where responders worked in Incident Management System leadership positions and as subject matter experts. The GRRT primary focus is international response, and responders are trained to work within varying cultural and environmental conditions outside the continental United States; however, the needs for assistance resulted in ≈90% of GRRT response time occurring in affected US territories and freely associated states. The GRRT roster, searchable by technical and language skills, facilitated the rapid identification of appropriate responders to fill response needs, particularly for speakers of Spanish and Portuguese, key languages in many of the affected areas. Although WHO declared the end of the emergency in November 2016 (*27*), GRRT will support CDC Zika virus response activities until no longer needed.

### 2016 Yellow Fever Response

In January 2016, the Angola Ministry of Health alerted WHO of an urban outbreak of yellow fever in Luanda Province (*28*). Because of active cross-border travel in the region, yellow fever cases spread to neighboring Democratic Republic of the Congo (DRC). In March 2016, the DRC Ministry of Health notified WHO of another yellow fever outbreak.

The CDC GDDOC closely monitored the evolution of the outbreak and coordinated mobilization needs with GRRT. Traditionally, the CDC response to a request for support would be led directly by subject matter experts; however, at the time, these experts were already fully engaged in the CDC Zika virus response and had limited capacity to lead another vectorborne disease response. Therefore, GRRT, in close coordination with GDDOC and anchored by expert guidance from CDC subject matter experts, contributed to the requested technical assistance and surge presence in the field.

During April–November 2016, GRRT mobilized 15 responders to Angola for 742 person-days and 7 responders to DRC for 211 person-days. Responders, working closely with expert guidance from headquarters, provided epidemiologic and management support to country ministries of health; led the interagency Incident Management System in the field on behalf of WHO; led field investigations and epidemiologic surveillance activities; and supported logistical needs, border health assessments, and a mass vaccination campaign. Four responders were mobilized to WHO headquarters to coordinate with and support the WHO yellow fever outbreak response. By August 2016, the last confirmed cases of yellow fever were reported, and the disease did not spread to additional countries. The last GRRT mobilization ended in November 2016.

The yellow fever response highlighted the benefits of agency surge capacity, particularly when specialized technical expertise is needed for multiple responses in multiple locations. The response also underscored the benefits of accurately identifying responders with high-level foreign language fluency but demonstrated the need to strengthen language capacity. Fluent speakers of Portuguese and French were identified for mobilization to Angola and DRC, respectively. However, because insufficient numbers of Portuguese speakers were available, fluent Spanish speakers partially filled the language gap.

### 2016 Haiti Hurricane Matthew Response

On October 4, 2016, Hurricane Matthew, a category 4 storm, made landfall in southwestern Haiti, causing major damage and flooding, killing at least 540 persons, and displacing ≈175,000 persons (*29*,*30*). Torrential rains washed away roads, bridges, and crops, threatening food security, water safety, telecommunication capabilities, and medical services (*29*). The hurricane devastated healthcare facilities, including 46 cholera treatment centers (*29*), and disrupted key public health programs.

After the 2010 earthquake in Haiti, GRRT surge staff, particularly ERRB responders, had experience in Haiti, and a field response was coordinated with the CDC Haiti Country Office. Because the CDC EOC was already coordinating 3 simultaneous activations for Ebola, Zika virus, and polio, GRRT and ERRB implemented the Incident Management System in the field and in ERRB workspace at CDC headquarters. Simultaneously, the CDC National Center for Environmental Health activated an Incident Management System to coordinate the domestic response for the expected effects to the US coastline. To foster coordination within the agency, both activations, outside of the CDC EOC, were supported by DEO in the early phases of the response.

GRRT mobilized the first wave of responders to Haiti 2 days after the hurricane struck. In total, GRRT mobilized 31 responders to Haiti, 26 members to the Atlanta-based Incident Management System structure, and 2 liaisons to the US Agency for International Development Office of Foreign Disaster Assistance and the Pan American Health Organization. In total, 1,302 person-days were spent responding to Hurricane Matthew.

GRRT responders supported the response in a diversity of roles. Early in the response, while physical access to affected areas was still limited, GRRT members organized a rapid phone assessment to provide critical information on the current needs of affected populations. CDC responders partnered with the Haiti Ministry of Health to investigate cholera cases, assess damage to healthcare facilities, and reestablish affected disease surveillance systems. Atlanta-based support staff mobilized to the CDC Haiti Country Office to support the Incident Management System structure, enabling the Haiti-based staff to fulfill their regular duties. At CDC headquarters, responders worked as Incident Management System staff coordinating the agency response and information managers for the CDC Haiti Country Office.

The Hurricane Matthew response demonstrated successful coordination of international and domestic response activities across the agency without burdening EOC staff. The GRRT/ERRB Incident Management System deactivated in November 2016, and the last mobilization for the Hurricane Matthew response ended in December 2016. An after-action review was conducted to evaluate the response and improve GRRT processes for future activations.

## Lessons Learned

The lessons learned from the Ebola epidemic forced many national and international organizations to reevaluate their emergency response capacity and processes. At CDC, these lessons contributed to the development of GRRT, a cadre of highly trained and experienced staff members and resources that provide response and surge capacity for CDC international emergency response operations. GRRT dedicated response staff enable rapid and longer mobilizations to establish and sustain working relationships with governments and partner organizations and to improve continuity of response activities. The large roster of >400 team members fosters a diversity of skills and experiences, and tracking of team member profiles facilitates matching technical skills and language capacity with response needs. GRRT support for CDC staff preparation and deployment readiness improves the speed at which qualified responders can be mobilized. GRRT capacity-building activities support countries’ progress toward IHR 2005 compliance, particularly around workforce development, personnel deployment, and emergency operations, in alignment with DEO and subject matter expert activities for other action packages.

Despite progress, several challenges remain. The Zika virus and yellow fever responses highlighted the need for strengthened language capacity. GRRT language training and targeted recruitment of highly proficient staff aim to address this gap; other language training options are being explored. CDC response capacity can be developed further by providing additional disease-specific technical training, particularly for high-risk pathogens and epidemic-prone diseases that may warrant a large-scale response. This training will build disease-specific response capacity and enable a limited set of subject matter experts to guide response activities in multiple areas, as was seen during the yellow fever response.

Moving forward, GRRT continues to evolve and seek new ways to improve international response capacity in coordination with international partners. Ongoing identification and rostering of responders with appropriate technical and language skills to fill response needs is critical for rapid response. The GRRT surge capacity roster will need to be maintained to keep responder information current and replenished with future qualified staff. CDC response mechanisms can be further improved through continued coordination with agency emergency response personnel and streamlined mobilization processes. To ensure a cohesive approach, GRRT will continue coordinating with external partners during emergency responses by identifying clear roles and responsibilities for staff (*20*). In addition, GRRT will continue supporting Global Health Security Agenda activities; building local, national, and regional response capacities; and supporting WHO, GOARN, and other international partners in global efforts toward development of international and regional public health rapid response teams. The lessons learned from the establishment of GRRT at CDC can serve as a model for the creation of similar response units in other countries.

## Conclusions

The CDC GRRT was established to address lessons learned during the 2014–2016 Ebola epidemic. Since June 2015, GRRT has been actively engaged in strengthening agency and partner emergency response capacity by developing a capable emergency workforce. However, continuing these activities and sustaining the momentum of global health security requires ongoing resources to ensure that GRRT is ready to respond to future health threats. CDC is one of many global organizations that respond to outbreaks and emergencies; no one organization alone can effectively control global health threats. As the international emergency response community coordinates to build capacity around the world, GRRT will work diligently so that disease threats are rapidly detected, responded to, and controlled at their source, thereby ensuring global health security.

## References

[1] Bell BP, Damon IK, Jernigan DB, Kenyon TA, Nichol ST, O’Connor JP, et al. Overview, control strategies, and lessons learned in the CDC response to the 2014–2016 Ebola epidemic. MMWR Suppl. 2016;65:4–11. 10.15585/mmwr.su6503a2 27389903

[2] Breakwell L, Gerber AR, Greiner AL, Hastings DL, Mirkovic K, Paczkowski MM, et al. Early identification and prevention of the spread of Ebola in high-risk African countries. MMWR Suppl. 2016;65:21–7. 10.15585/mmwr.su6503a4 27389301

[3] Frieden TR. Foreword. MMWR Suppl. 2016;65:1–3. 10.15585/mmwr.su6503a1 26916400

[4] Centers for Disease Control and Prevention. Outbreaks chronology: Ebola virus disease [cited 2017 Feb 24]. http://www.cdc.gov/vhf/ebola/outbreaks/history/chronology.html

[5] Centers for Disease Control and Prevention. 2014 Ebola outbreak in West Africa–case counts [cited 2017 Mar 2]. https://www.cdc.gov/vhf/ebola/outbreaks/2014-west-africa/case-counts.html

[6] Centers for Disease Control and Prevention. Why global health security matters [cited 2017 Feb 24]. https://www.cdc.gov/globalhealth/security/why.htm

[7] Heymann DL, Chen L, Takemi K, Fidler DP, Tappero JW, Thomas MJ, et al. Global health security: the wider lessons from the west African Ebola virus disease epidemic. Lancet. 2015;385:1884–901. 10.1016/S0140-6736(15)60858-3 25987157 PMC5856330

[8] Moon S, Leigh J, Woskie L, Checchi F, Dzau V, Fallah M, et al. Post-Ebola reforms: ample analysis, inadequate action. BMJ. 2017;356:j280. 10.1136/bmj.j280 28115316

[9] Gostin LO, Tomori O, Wibulpolprasert S, Jha AK, Frenk J, Moon S, et al. Toward a common secure future: four global commissions in the wake of Ebola. PLoS Med. 2016;13:e1002042. 10.1371/journal.pmed.1002042 27195954 PMC4873000

[10] Mackey TK. The Ebola outbreak: catalyzing a “shift” in global health governance? BMC Infect Dis. 2016;16:699. 10.1186/s12879-016-2016-y 27881085 PMC5121963

[11] World Health Organization. International Health Regulations. 2nd ed. Geneva: The Organization; 2005.

[12] Braden CR, Dowell SF, Jernigan DB, Hughes JM. Progress in global surveillance and response capacity 10 years after severe acute respiratory syndrome. Emerg Infect Dis. 2013;19:864–9. 10.3201/eid1906.130192 23731871 PMC3713843

[13] Centers for Disease Control and Prevention. Global health security agenda: action packages [cited 2017 Mar 2]. https://www.cdc.gov/globalhealth/security/actionpackages/default.htm

[14] Frieden TR, Tappero JW, Dowell SF, Hien NT, Guillaume FD, Aceng JR. Safer countries through global health security. Lancet. 2014;383:764–6. 10.1016/S0140-6736(14)60189-6 24529561 PMC7133716

[15] Centers for Disease Control and Prevention. The global health security agenda [cited 2017 Feb 24]. https://www.cdc.gov/globalhealth/security/ghsagenda.htm

[16] Centers for Disease Control and Prevention. CDC’s role in global health security [cited 2017 Mar 2]. https://www.cdc.gov/globalhealth/security/cdcrole.htm

[17] Centers for Disease Control and Prevention. Global Disease Detection (GDD) Operations Center [cited 2017 Mar 2]. https://www.cdc.gov/globalhealth/healthprotection/gddopscenter/index.html

[18] Centers for Disease Control and Prevention. Office of Public Health Preparedness and Response: overview [cited 2017 Mar 2]. https://www.cdc.gov/phpr/about.htm

[19] Rouse EN, Zarecki SM, Flowers D, Robinson ST, Sheridan RJ, Goolsby GD, et al. Safe and effective deployment of personnel to support the Ebola response—West Africa. MMWR Suppl. 2016;65:90–7. 27387289 10.15585/mmwr.su6503a13

[20] Dahl BA, Kinzer MH, Raghunathan PL, Christie A, De Cock KM, Mahoney F, et al. CDC’s r to the 2014–2016 Ebola epidemic—Guinea, Liberia, and Sierra Leone. MMWR Suppl. 2016;65:12–20. 10.15585/mmwr.su6503a3 27388930

[21] Frieden TR, Damon IK. Ebola in West Africa—CDC’s role in epidemic detection, control, and prevention. Emerg Infect Dis. 2015;21:1897–905. 10.3201/eid2111.150949 26484940 PMC4622264

[22] Arwady MA, Bawo L, Hunter JC, Massaquoi M, Matanock A, Dahn B, et al. Evolution of ebola virus disease from exotic infection to global health priority, Liberia, mid-2014. Emerg Infect Dis. 2015;21:578–84. 10.3201/eid2104.141940 25811176 PMC4378496

[23] Brooks JC, Pinto M, Gill A, Hills KE, Murthy S, Podgornik MN, et al. Incident management systems and building emergency management capacity during the 2014–2016 Ebola epidemic— Liberia, Sierra Leone, and Guinea. MMWR Suppl. 2016;65:28–34. 10.15585/mmwr.su6503a5 27389463

[24] World Health Organization. Joint External Evaluation tool: International Health Regulations (2005) [cited 2017 Mar 2]. http://www.who.int/iris/handle/10665/204368

[25] Pan American Health Organization. Epidemiological Alert: neurological syndrome, congenital malformations, and Zika virus infection. Implications for public health in the Americas, 1 December 2015 [cited 2017 Mar 27]. http://www2.paho.org/hq/index.php?option=com_docman&task=doc_view&Itemid=270&gid=32405&lang=en

[26] Oussayef NL, Pillai SK, Honein MA, Ben Beard C, Bell B, Boyle CA, et al. Zika virus—10 public health achievements in 2016 and future priorities. MMWR Morb Mortal Wkly Rep. 2017;65:1482–8. 10.15585/mmwr.mm6552e1 28056005

[27] World Health Organization. Fifth meeting of the Emergency Committee under the International Health Regulations (2005) regarding microcephaly, other neurological disorders and Zika virus [cited 2017 Mar 2]. http://www.who.int/mediacentre/news/statements/2016/zika-fifth-ec/en/

[28] World Health Organization. Yellow fever—Angola [cited 2017 Mar 2]. http://www.who.int/csr/don/14-june-2016-yellow-fever-angola/en/

[29] United Nations Office for the Coordination of Humanitarian Affairs. Haiti: Hurricane Matthew, situation report no. 20 (8 November 2016) [cited 2017 Mar 2]. http://reliefweb.int/sites/reliefweb.int/files/resources/sitrep_20_-_haiti_08_nov_2016_-_en.pdf

[30] United Nations Office for the Coordination of Humanitarian Affairs. Haiti: Hurricane Matthew, situation report no. 25 (25 November 2016) [cited 2017 Mar 2]. http://reliefweb.int/sites/reliefweb.int/files/resources/OCHA%20Situation%20Report%20%2325%20Hurricane%20Matthew%20Haiti%2025%20Nov%202016%20FINAL.pdf

